# Characterization of HIV-1 Epidemic in Kyrgyzstan

**DOI:** 10.3389/fmicb.2021.753675

**Published:** 2021-10-15

**Authors:** Mariya V. Sivay, Alexei V. Totmenin, Daria P. Zyryanova, Irina P. Osipova, Tatyana M. Nalimova, Mariya P. Gashnikova, Vladimir V. Ivlev, Ivan O. Meshkov, Umut Z. Chokmorova, Elmira Narmatova, Ulukbek Motorov, Zhyldyz Akmatova, Nazgul Asybalieva, Aybek A. Bekbolotov, Ulan K. Kadyrbekov, Rinat A. Maksutov, Natalya M. Gashnikova

**Affiliations:** ^1^Department of Retroviruses, State Research Center of Virology and Biotechnology “Vector”, Koltsovo, Russia; ^2^National Research Center for Hematology, Moscow, Russia; ^3^Republican Center of AIDS, Ministry of Health of Kyrgyzstan, Bishkek, Kyrgyzstan; ^4^Osh Regional Center of AIDS Treatment and Prevention, Osh, Kyrgyzstan

**Keywords:** HIV phylogenetics, phylodynamic analysis, Kyrgyzstan, Central Asia, HIV molecular epidemiology

## Abstract

Kyrgyzstan has one of the highest rates of HIV-1 spread in Central Asia. In this study, we used molecular–epidemiological approaches to examine the HIV-1 epidemic in Kyrgyzstan. Samples were obtained from HIV-positive individuals who visited HIV/AIDS clinics. Partial *pol* gene sequences were used to identify HIV-1 subtypes and drug resistance mutations (DRMs) and to perform phylogenetic analysis. Genetic diversity and history reconstruction of the major HIV-1 subtypes were explored using BEAST. This study includes an analysis of 555 HIV-positive individuals. The study population was equally represented by men and women aged 1–72 years. Heterosexual transmission was the most frequent, followed by nosocomial infection. Men were more likely to acquire HIV-1 during injection drug use and while getting clinical services, while women were more likely to be infected through sexual contacts (*p* < 0.01). Heterosexual transmission was the more prevalent among individuals 25–49 years old; individuals over 49 years old were more likely to be persons who inject drugs (PWID). The major HIV-1 variants were CRF02_AG, CRF63_02A, and sub-subtype A6. Major DRMs were detected in 26.9% of the study individuals; 62.2% of those had DRMs to at least two antiretroviral (ARV) drug classes. Phylogenetic analysis revealed a well-defined structure of CRF02_AG, indicating locally evolving sub-epidemics. The lack of well-defined phylogenetic structure was observed for sub-subtype A6. The estimated origin date of CRF02_AG was January 1997; CRF63_02A, April 2004; and A6, June 1995. A rapid evolutionary dynamic of CRF02_AG and A6 among Kyrgyz population since the mid-1990s was observed. We observed the high levels of HIV-1 genetic diversity and drug resistance in the study population. Complex patterns of HIV-1 phylogenetics in Kyrgyzstan were found. This study highlights the importance of molecular–epidemiological analysis for HIV-1 surveillance and treatment implementation to reduce new HIV-1 infections.

## Introduction

Kyrgyzstan, or Kyrgyz Republic, is a small country in Central Asia bordering with Kazakhstan, Uzbekistan, Tajikistan, and China. In 2020, an estimated 9,200 (8,400--9,900) people lived with HIV-1 in Kyrgyzstan with a prevalence of 0.2%^1^. The HIV-1 epidemic concentrates in the key populations, mainly persons who inject drugs (PWID) and their sexual partners (Zhao et al., 2020). Access to HIV-1 care and treatment services is far behind the global target of 95-95-95 by 2030. In 2020, 76% of people were aware of their HIV-1 status, 48% of them received antiretroviral therapy (ART), and only 43% of those were viral suppressed; the ART coverage among pregnant women was 94% (see text footnote 1).

Molecular epidemiology and phylogenetic analysis are widely used to characterize HIV-1 epidemics. HIV-1 phylogenetics has been extensively used to characterize the virus transmission networks (Wilkinson et al., 2014; Grabowski et al., 2018) and to reconstruct the viral origin and spread history (Wilkinson et al., 2014). The combination of epidemiological and sociodemographic data with the phylogenetic approaches reveals data on the risk factors associated with the HIV-1 spread (Wolf et al., 2017; Fujimoto et al., 2021), identifies sub-epidemics (Peters et al., 2016), and informs prevention and care interventions (Brenner and Wainberg, 2013).

Molecular–epidemiological and phylogenetic studies of HIV-1 infection in Central Asia, particularly in Kyrgyzstan, are very limited. One of the most recent studies in Kyrgyzstan describes a high rate (range 39–50%) of HIV-1 drug resistance in individuals with treatment failure (Lapovok et al., 2020). The study of HIV-1 sub-subtype A6 [also known as Russian A1 or A-FSU (Foley et al., 2016)] describing the transmission networks in former Soviet Union (FSU) countries concludes that the major driving source of HIV-1 transmission is migrant workers, emphasizing an extensive mobility in the region (80–90% of migrants are from Central Asia) (Aibekova et al., 2018). The study also points at the emerging HIV-1 epidemic among hetero- and homosexual populations, surpassing the parenteral transmission (Aibekova et al., 2018). Drug trafficking from Afghanistan and Tajikistan to southern Kyrgyzstan significantly increased in the mid-1990s, resulting in predominant opiate injections over the homemade drugs (Wolf et al., 2008). Between 1991 and 1999, registered drug use rate in Kyrgyzstan increased sevenfold (Wolf et al., 2008). And in the following 5-year period from 2001 to 2006, Kyrgyzstan had a 15-fold increase of HIV-1 infections, with 76% of cases detected among PWID (Wolf et al., 2008). However, in the last decade, national programs on HIV/AIDS in cooperation with international programs (Thorne et al., 2010; Zhao et al., 2020) have significantly improved HIV-1 care services in the country. While in 2010, only 9% of HIV-infected people received ART, this indicator reached 48% in 2020. In 2010, only 17% HIV-infected people on ART were virally suppressed; this number almost tripled by 2020 (see text footnote 1). By the beginning of 2021, 86.5% of HIV-positive individuals on ART had undetectable viral loads^2^.

In this study, we performed the HIV-1 molecular–epidemiological survey in Kyrgyzstan. To achieve that goal, we analyzed HIV-1 genetic diversity and HIV-1 drug resistance, identified potential transmission clusters, described epidemiological characteristics of studied individuals, and reconstructed the evolutionary history of the virus.

## Materials and Methods

### Study Population

The blood samples were collected from HIV-positive adults and children who visited local HIV/AIDS clinics of the Ministry of Health of Kyrgyzstan. All the individuals in this study were diagnosed with HIV-1 infection a year prior to the sample collection and were on ART for at least a year. Samples were collected in four provinces (Bishkek, Osh, Jalal-Abad, and Batken) in Kyrgyzstan from 2016 to 2019. Data collected from individuals in Jalal-Abad and Batken were combined (further denoted as JAB) due to the small sample size and geographical closeness of these regions. HIV-1 testing was performed at the study sites according to the national guideline^3^. Samples were shipped to the Department of Retroviruses, State Research Center of Virology and Biotechnology “Vector” (Koltsovo, Novosibirsk region, Russia) for further testing. Demographic and HIV-related characteristics of individuals were collected at the local healthcare facilities from clinical records. A woman was assigned a “pregnant” status if she was pregnant at the time of her visit for the sample collection. In this study, we refer “children” to individuals 14 years of age and under; “young adults,” between 15 and 24 years old; “adults,” between 25 and 49 years old; and “older adults,” individuals of 50 years of age and above. The study did not recruit individuals from any group; only individuals who visited HIV/AIDS clinics were included. However, samples from children and young adults who had in-hospital acquired HIV-1 infection were particularly collected to investigate those cases.

### Amplification of HIV-1 Pol Gene Fragment and Sequencing Analysis

Viral RNA or proviral DNA was extracted using the RealBest DeltaMag kit (Vector-Best, Novosibirsk, Russia) according to the manufacturer’s manual. RNA/DNA was used for the amplification of HIV-1 partial *pol* gene region coding protease and reverse transcriptase (HXB2 #K03455 reference strain coordinates: 2249–3420). Amplification of the *pol* gene fragments and sequencing analysis were performed as previously described (Maksimenko et al., 2020).

### HIV-1 Subtyping and Drug Resistance Analysis

HIV-1 subtyping was performed using automated HIV-1 subtyping tools REGA v 3.0 (Pineda-Pena et al., 2013), COMET (Struck et al., 2014), and recombinant identification program (RIP) (Siepel et al., 1995). HIV-1 subtypes were also investigated using an approximately maximum-likelihood phylogenetic method using FastTree v2.1.9 (Price et al., 2010) with HIV-1 subtype references from Los Alamos National Laboratory (LANL) HIV Sequence Database, 2020^4^. HIV-1 subtype was assigned if three out of four methods agree. Drug resistance mutations (DRMs) were assessed using Stanford HIV drug resistance database (HIVdb Program) (Shafer, 2006). DRM was considered as major according to Stanford HIV drug resistance database.

### Phylogenetic and Cluster Analyses

Phylogenetic analysis was conducted for study and background sequences; background sequences were selected from BLAST as 100 most closely related (with a BLAST score < 1e–50) to each study sequence. Sequences were aligned using MAFFT software (Katoh et al., 2017). Recombination analysis was performed for study and background sequences using RDP4 (Martin et al., 2015). Sites of major DRMs (43 codon positions) were removed. Phylogenetic trees were constructed using IQ-TREE v2 (Minh et al., 2020) under the GTR + G4 + I substitution model. Phylogenetic trees were visualized using interactive Tree of Life (iTOL) (Letunic and Bork, 2021). Monophyletic groups of study sequences with branch support ≥ 80 were considered as a distinct viral lineage (clades). Transmission clusters (≥2 individuals infected by direct/indirect transmission of genetically related HIV-1 variants) were identified by Cluster Picker v1.2.3 (Ragonnet-Cronin et al., 2013) using thresholds of 0.045 of maximum pairwise genetic distance between sequences and a branch support of 90.

### Phylodynamic Analysis

Genetic diversity and history reconstruction of the major HIV-1 subtypes were explored using a Bayesian Markov chain Monte Carlo (MCMC) phylogenetic analysis using the BEAST v1.10.4 (Suchard et al., 2018). The temporal structure of the datasets was estimated using TempEst v1.5.3 (Rambaut et al., 2016). Analysis was performed using GTR + G4 + I substitution model, with different combinations of molecular clock models (strict and log-normal uncorrelated relaxed), and coalescent models [constant size, exponential growth, Bayesian Skyline, and Gaussian Markov random field (GMRF) Bayesian Skyride]. The adjustment to the data was estimated using the log marginal likelihood estimation (MLE) using path sampling/stepping-stone sampling (PS/SS). The best-fit model was selected based on the Bayes factor (BF; BF ≥ 3 was considered significant). Two independent MCMC runs were performed for 70 × 10^6^ generations. Convergence of the chains was estimated based on the effective sampling size (ESS; cutoff value over 200 for all the parameters) in Tracer v1.7.1 (Rambaut et al., 2018).

### Statistical Analysis

Categorical variables were analyzed using modified Fisher’s test; quantitative variables were analyzed using the Kruskal–Wallis test. The Monte Carlo method (10^6^ simulations) was used for p-value (P) estimation. P correction was performed to control the false discovery rate using the Benjamini–Yekutieli procedure. Statistical analysis was performed in RStudio Team (2020) v1.1.422^5^.

### Nucleotide Sequence Accession Numbers

Study sequences were submitted to GenBank under accession numbers MG798935--MG799123, MK228729--MK228833, and MW303524--MW303757 and can be found in the online repository^6^.

### Ethics Statement

The study was approved by the Ethical Committee of Research and Manufacturing Association “Prevention Medicine” of Ministry of Health of Kyrgyz Republic (2017). Written consent forms were provided by all the study individuals. For individuals 18 years old and younger, written consent forms were provided by their parents or legal guardians.

## Results

### Study Population

The study population included 555 individuals who resided in four Kyrgyz provinces (202 individuals from Bishkek, 341 individuals from Osh, and 12 individuals from JAB). Detailed characteristics of the study individuals are presented in Table 1 and Supplementary Table. Men and women were equally represented in the study population (50.8% men vs. 49.2% women), with a median age of 31 years (range 3–72 years old). HIV-1 infection prevailed among individuals in the 25–49 age group. The median time since HIV-1 diagnosis was 8.1 years (range 1–19 years); the median time since HIV-1 diagnosis was longer in Osh than in Bishkek (9 vs. 6.46 years, *p* < 0.01). Heterosexual transmission was the most prevalent (38.7%), followed by the nosocomial infection (21.4%). In Bishkek, over a half of individuals were infected through heterosexual contacts, and 30% were PWID. In Osh, most of the individuals acquired HIV-1 while getting clinical services. Men were more likely to acquire HIV-1 infection during drug injections (33.5%) and clinical services (25.3%), while women were more likely acquire HIV-1 infection through sexual contacts (72.6%, *p* < 0.01). When stratified by age group, heterosexual transmission was more prevalent among adults; older adults were more likely to acquire HIV-1 while injecting drugs. All the individuals with nosocomial HIV-1 infection were 24 years old and younger. HIV-1 DRMs to at least two ARV drug classes were more frequent in children and young adults compared with older groups (*p* < 0.01). Individuals 24 years of age and younger tended to have HIV-1 infection caused by CRF02_AG, while older individuals were more likely to be infected with sub-subtype A6 (*p* < 0.01). Nine (1.6%) individuals were identified as men who have sex with men (MSM), with a median age of 28 years (range 23–37 years old); seven of those individuals resided in Bishkek province. The median time since HIV-1 diagnosis was 4.6 years (range 4–6 years). Four (44.4%) of nine MSM were infected by minor HIV-1 variants (subtype B, subtype G, URF B/CRF02_AG, and URF B/G); the remaining five MSM had sub-subtype A6 (*n* = 4) and CRF02_AG (*n* = 1) HIV-1 infection. None of MSM had HIV-1 DRMs detected.

**TABLE 1:** Demographic and HIV-related characteristics of the 555 study individuals.

Characteristics	Total, *n* = 555	Bishkek, *n* = 202	Osh, *n* = 341	JAB, *n* = 12	*p*	Adj. *p*
**Gender, *n* (%)**						
Male	282 (50.8)	98 (48.5)	180 (52.8)	4 (33.3)	0.41	1
Female	273 (49.2)	104 (51.5)	161 (47.2)	8 (66.7)		
**Age (years), *n* (%)**						
3–14	101 (18.2)	5 (2.5)	95 (27.9)	1 (8.3)	**< 0.01**	**< 0.01**
15–24	120 (21.6)	17 (6.9)	103 (30.2)	–		
25–49	271 (48.8)	143 (71.1)	119 (34.9)	7 (66.7)		
50–72	63 (11.4)	36 (17.9)	24 (7)	3 (25)		
Years since HIV-1 diagnosis, median	8.1 (range: 1–19)	6.46 (range: 1–18)	9 (range: 2–19)	8.92 (range: 3–15)	**< 0.01**	**< 0.01**
**Transmission mode, n (%)**						
Heterosexual	215 (38.7)	117 (57.9)	90 (26.4)	8 (66.7)	**< 0.01**	**< 0.01**
MSM	9 (1.6)	7 (3.5)	2 (0.6)	–		
Vertical	70 (12.6)	8 (4)	60 (17.9)	1 (8.3)		
PWID	103 (18.6)	61 (30.2)	40 (11.7)	3 (25)		
Nosocomial	119 (21.4)	–	119 (34.9)	–		
Unknown/No data	39 (7)	9 (4.5)	30 (8.8)	–		
Pregnancy status, *n* (%) Pregnant	33 (12.1)	33 (31.7)	–	–	–	–
**HIV-1 drug resistance mutations, *n* (%)**						
Yes No	149 (26.9) 406 (73.1)	50 (24.8) 152 (75.2)	97 (28.5) 245 (71.5)	2 (16.7) 9 (83.3)	0.3	0.91
**Dual- and multi-class HIV-1 drug resistance, *n* (%)**						
Yes No	99 (66.4) 50 (33.6)	29 (56.9) 21 (43.1)	70 (72.2) 27 (27.8)	0 11 (100)	0.06	0.68
**HIV-1 subtyping, *n* (%)**						
CRF02_AG	332 (59.8)	95 (47)	232 (68)	5 (41.7)	**< 0.01**	**< 0.01**
A6	184 (33.2)	90 (44.6)	89 (26.1)	5 (41.7)		
CRF63_02A	10 (1.8)	4 (2)	4 (1.2)	2 (16.6)		
Minor subtypes and recombinants	29 (5.2)	13 (6.4)	16 (4.7)	–		

*Numbers in bold indicate statistically significant associations. JAB, Jalal-Abad and Batken provinces; Adj. *p*, adjusted p-value; MSM, men who have sex with men; PWID, persons who inject drugs; CRF, circulating recombinant form.*

### HIV-1 Subtyping and Drug Resistance

Genotyping results were successfully obtained for 555 (89.4%) of 621 individuals. The most frequent HIV-1 subtypes were CRF02_AG [*n* = 332 (59.8%)], CRF63_02A [*n* = 10 (1.8%)], and sub-subtype A6 [*n* = 184 (33.2%)]. The remaining 29 (5.2%) sequences represent the minor HIV-1 subtypes and unique recombinant forms (URFs). Detailed HIV-1 subtype distribution is shown in Table 2.

**TABLE 2:** Distribution of HIV-1 subtypes by the study provinces.

Subtype, n (%)	Bishkek, *n* = 202	Osh, *n* = 341	JAB, *n* = 12
CRF02_AG	95 (47%)	232 (68%)	5 (41.7%)
A6	90 (44.6%)	89 (26.1%)	5 (41.7%)
CRF63_02A	4 (2%)	4 (1.8%)	2 (16.6%)
A6 URFs	3 (1.5%)	5 (1.5%)	
G	3 (1.5%)	2 (0.6%)	
B	3 (1.5%)	2 (0.6%)	
CRF02_AG/A6	2 (1%)	4 (1.8%)	
CRF02_AG/B	1 (0.5%)	1 (0.3%)	
A6/G	–	1 (0.3%)	
B/G	1 (0.5%)	1 (0.3%)	

*CRF, circulating recombinant form; URF, unique recombinant form; JAB, Jalal-Abad and Batken provinces.*

**FIGURE 1:**
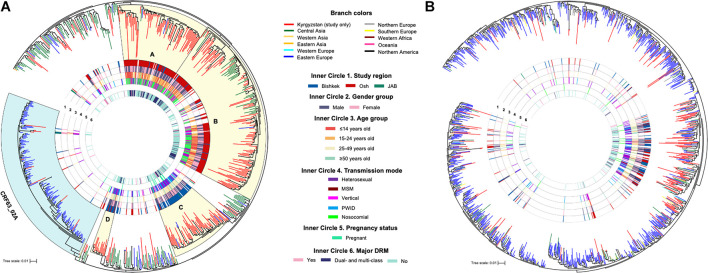
Maximum-likelihood phylogenetic trees of HIV-1 *pol* sequences of CRF02_AG/CRF63_02A **(A)** and sub-subtype A6 **(B)**. Monophyletic clades (A–D) with the branch support ≥ 80 and containing for over 70% of study sequences are shaded in yellow. JAB, Jalal-Abad and Batken provinces; CRF, circulating recombinant form; MSM, men who have sex with men; PWID, persons who inject drugs; DRM, drug resistance mutation.

Major DRMs were identified in 149 of 555 (26.9%) sequences (Table 2 and Figure 1). Nucleoside reverse-transcriptase inhibitor (NRTI)-resistance mutations were detected in 107 individuals. Non-NRTI (NNRTI)-resistance mutations were detected in 138 individuals. Protease inhibitor (PI)-resistance mutations were detected in four individuals. Resistance mutations to at least two ARV drug classes were identified in 99/149 (66.4%) sequences.

### Phylogenetic and Cluster Analyses

Phylogenetic trees were constructed for each major HIV-1 subtype separately (Figure 1); sequences of CRF02_AG and CRF63_02A were combined, and a single phylogenetic tree was constructed. Nineteen study sequences (CRF02_AG, *n* = 11 and A6, *n* = 8) were excluded from the analysis due to short sequence length (less than 1,000 base pairs). Final datasets included 325 study and 486 background sequences of CRF02_AG/CRF63_02A, and 173 study and 1,056 background sequences of sub-subtype A6. Phylogenetic analyses of CRF02_AG/CRF63_02A and sub-subtype A6 datasets revealed that Kyrgyz sequences intermingle with sequences from the neighboring countries, indicating genetic similarity and the potential common origins with corresponding variants in the Eastern Europe and Central Asia regions. The CRF02_AG/CRF63_02A tree shows the presence of the four well-defined clades composed of the local study sequences; two of those clades (A and B, 97 and 100 branch support values) are predominantly represented by the study sequences from Bishkek (Figure 1A). Two other clades (C and D, branch support values of 84 and 87) primarily include study sequences from Osh. This possibly represents the four distinct locally evolving sub-epidemics of CRF02_AG in Kyrgyzstan. Clades A and B were mainly represented by individuals aged 25–49 years infected by heterosexual and PWID transmission modes. Clades C and D are predominantly presented by children and young adults with the nosocomial HIV-1 infection. No noticeable clades were revealed among sub-subtype A6 study sequences; study samples scattered across the background sequences, indicating multiple independent introduction events of sub-subtype A6 to Kyrgyzstan from the neighboring countries (Figure 1B).

**FIGURE 2:**
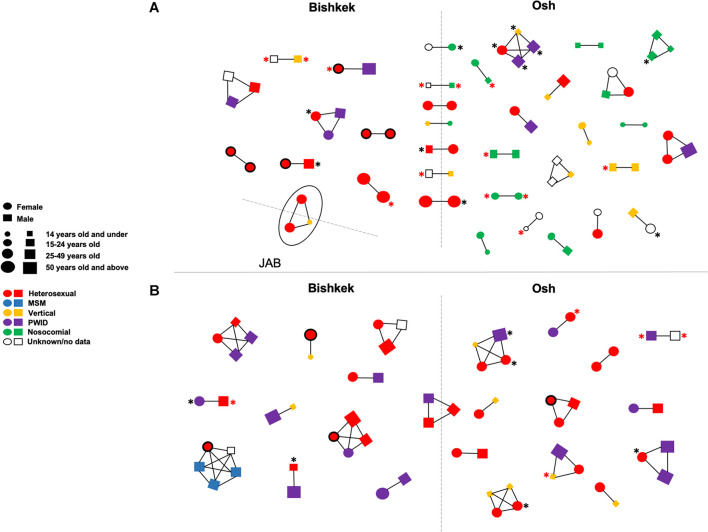
Transmission clusters of study HIV-1 *pol* sequences ofCRF02_AG/CRF63_02A **(A)** and sub-subtype A6 **(B)**. Clusters were detected in IQ-TREE at maximum 0.045 genetic distances and 90 branch support thresholds. Each figure (circle/square) corresponds to a node on the phylogenetic tree; figure shape, size, and color correspond to an individual’s gender, age, and HIV-1 transmission mode according to the left center footnote. The line drawn between figures indicates that HIV-1 sequences from the respective individuals in phylogenetic tree fell within the clustering thresholds. The black asterisk indicates the HIV-1 drug resistance; the red asterisk indicates the HIV-1 drug resistance to at least two ARV drug classes. Circles with the bold black border correspond to the pregnant women. The solid oval corresponds to a cluster of three individuals infected by CRF63_02A. The dashed lines divide figures corresponding to individuals from the different study regions. JAB, Jalal-Abad and Batken provinces; MSM, men who have sex with men; PWID, persons who inject drugs.

Fifty-seven putative transmission clusters representing 140 individuals (28.1%; 140/498) were detected; 34 clusters were identified among CRF02_AG sequences, 23 clusters among A6 sequences, and one cluster of CRF63_02A sequences (Figure 2). Most of the clusters were pairs (*n* = 38) and triplets (*n* = 12). Five clusters of four and one cluster of five sequences were also found. Nine clusters were identified among individuals from different study regions. Major DRMs were detected in 33 (23.6%) clustered individuals; 17 of those individuals had DRMs to at least two ARV dug classes. No statistically significant association between demographic and HIV-related characteristics of the study individuals and phylogenetic clustering was found.

### Phylodynamic Analysis

**FIGURE 3:**
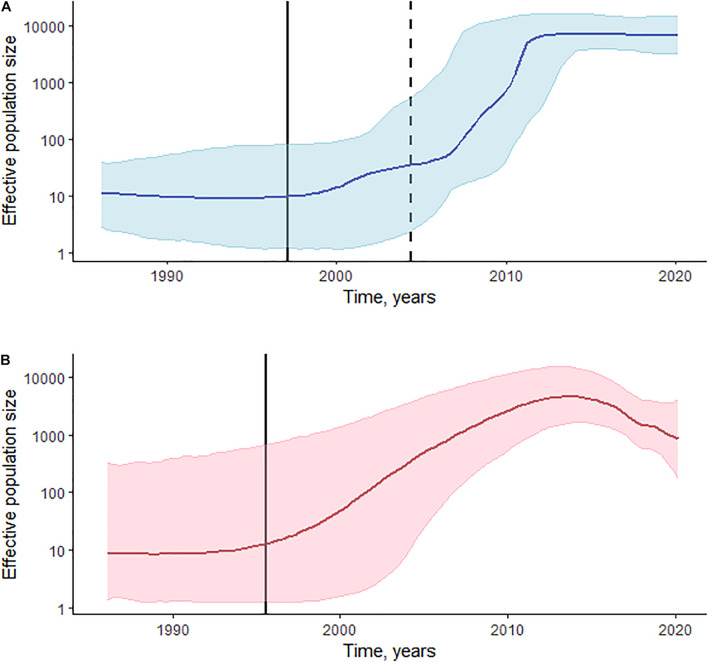
Phylodynamic reconstruction of HIV-1 sCRF02_AG/CRF63_02A **(A)** and sub-subtype A6 **(B)** in Kyrgyzstan. **(A)** The Bayesian Skyline plot was reconstructed for the 325 *pol* gene sequences of CRF02_AG and 10 sequences of CRF63_02A. The horizontal bold line indicates effective population size through time; blue-shaded area represents the 95% highest posterior density (HPD). The vertical bold black line indicates the estimated origin date for CRF02_AG [January 1997 (95% HPD: February 1986–May 2004)]; the vertical dashed line indicates the estimated origin date for CRF63_02A [April 2004 (95% HPD: January 1997–April 2009)]. **(B)** The Gaussian Markov random field (GMRF) Bayesian Skyride plot was reconstructed for the 173 *pol* gene sequences of sub-subtype A6. The horizontal bold line indicates effective population size through time; pink-shaded area represents the 95% HPD. The vertical bold black line indicates the estimated origin date for sub-subtype A6 [June 1995 (95% HPD: August 1985–August 2002)].

Log-normal uncorrelated relaxed molecular clock model outperforms a strict model based on the BFs in both CRF02_AG/CRF63_02A and sub-subtype A6 datasets. BFs also indicate an advantage of the Bayesian Skyline coalescent model in CRF02_AG/CRF63_02A, and a small advantage of the Gaussian Markov random field (GMRF) Bayesian Skyride coalescent model in the A6 dataset. The estimated median substitution rate for CRF02_AG/CRF63_02A was 2.79 × 10^–3^ (95% highest posterior density [HPD]: 1.93 × 10^–3^–3.69 × 10^–3^), and 2.55 × 10^–3^ (95% HPD: 1.69 × 10^–3^–3.43 × 10^–3^) for sub-subtype A6. The demographic history of HIV-1 CRF02_AG/CRF63_02A and sub-subtype A6 in Kyrgyzstan is presented in Figure 3. CRF02_AG/CRF63_02A Skyline plot reveals a rapid growth phase in ESS between the late 1990s and 2010 followed by a stable phase (Figure 3A). The estimated date of origin of the CRF02_AG epidemic in Kyrgyzstan was January 1997 (95% HPD: February 1986–May 2004). The origin date of CRF63_02A in Kyrgyzstan was estimated as April 2004 (95% HPD: January 1997–April 2009). The origin dates for the four major CRF02_AG clades were estimated as September 2000 (95% HPD: May 1993–August 2005) for clade D, May 2003 (95% HPD: May 1966–March 2008) for clade C, 2005 (95% HPD: March 1999–February 2009) for clade A, and September 2006 (95% HPD: July 2001–May 2010) for clade B. The date of origin for sub-subtype A6 was estimated as June 1995 (95% HPD: August 1985–August 2002). Sub-subtype A6 Skyride plot had a growth phase in ESS in the mid-2010s; the growth rate began to decline somewhat thereafter (Figure 3B).

## Discussion

In this study, we performed molecular–epidemiological analysis of HIV-1 in Kyrgyzstan in four provinces, two of which (Bishkek and Osh) are most severely affected by HIV-1. Our results showed the predominant circulation of two major HIV-1 variants—CRF02_AG and sub-subtype A6. A third of HIV-infected individuals in the study had major HIV-1 DRMs: two-third of those had DRMs to at least two ARV drug classes. Phylogenetic analysis revealed a well-defined structure of CRF02_AG indicating the locally evolving sub-epidemics. The lack of a well-defined phylogenetic structure was observed for the A6 sub-subtype. Small distinct HIV-1 transmission clusters were detected among study sequences. Clustering was not associated with any individual characteristics. DRMs were detected in 26.9% of clustered individuals.

HIV-1 epidemic in Kyrgyzstan is characterized by an overall low HIV-1 infection prevalence rate, a high HIV-1 prevalence rate among key populations (Deryabina et al., 2019), and a high proportion of HIV-positive children (Mansfeld et al., 2015). Our study found that most of the HIV-positive older adults were PWID; heterosexual transmission was more common in the 25–49 age group. This may indicate the shift of the HIV-1 epidemic to the general population from key groups such as PWID or change of substance consumption to non-injection drugs. Our study also found that over a half of children and young adults in Osh acquired healthcare-associated HIV-1 infection. Earlier studies described several outbreaks of hospital-acquired HIV-1 among children in Osh province in 2007 (Thorne et al., 2010) and 2011–2012 (Mansfeld et al., 2015), with the total number of over 300 reported cases. Although healthcare-associated HIV-1 infections in high-income countries are extremely low, risk of nosocomial HIV-1 infections in the resource-limited countries remains high (Ganczak and Barss, 2008; Myburgh et al., 2020). We also identified significant differences in the transmission modes between genders. Women were more likely to be infected thought the sexual contacts; PWID was a dominant transmission mode among men, followed by nosocomial infection. Similar epidemiological characteristics of HIV-positive individuals were described in the previous report (Zhao et al., 2020).

The first HIV-1 case in Kyrgyzstan was identified in 1987 in a foreigner [34]. The first local HIV-1 case was registered in 1996 (Roth et al., 2012), and HIV-1 cases in Kyrgyzstan are steadily increasing since then, like in other countries of Central Asia and Eastern Europe. CRF02_AG predominantly circulates in West Africa (Bbosa et al., 2019), but since 1999, this recombinant form is constantly detected in Central Asian countries (Laga et al., 2015; Aibekova et al., 2018). CRF02_AG circulating in Central Asia is genetically distant from that from African countries and phylogenetically represents well-supported distant clade (Mir et al., 2016). Our study showed the presence of the four main CRF02_AG region-specific lineages circulating in Kyrgyzstan. Phylodynamic reconstruction of HIV-1 epidemics in Kyrgyzstan revealed the date of origin of CRF02_AG as January 1997. Osh-specific clades increased earlier than did the Bishkek-specific clades. Analysis of the characteristics of the study individuals also showed that people from Osh had HIV-1 infection for a longer period than those from Bishkek. The CRF02_AG rise in Osh coincided with the active drug trafficking from Afghanistan through the so-called ‘‘North route.’’ The main traffic routes go to the South Kyrgyzstan (Osh province) through the Tajikistan and Uzbekistan and then spread across Kyrgyzstan and further to Kazakhstan, Russian, and other European countries^7^. Most likely, this viral variant was introduced to Kyrgyzstan by drug trafficking [Mir et al., 2016]. Bishkek-specific clades were predominantly represented by adults who acquired HIV-1 infection through heterosexual contacts and while injection of drugs, indicating an extensive mixing between high-risk and heterosexual populations. Osh-specific clades are represented by children and young adults who acquired healthcare-associated HIV-1 infection and represented by in-hospital HIV-1 outbreak sequences. These age groups play an important role in the local HIV-1 sub-epidemic, and targeted HIV-1 care interventions should be implemented to limit HIV-1 spread and improve healthcare services. HIV-1 sub-subtype A6 epidemic in FSU countries is characterized by monophyletic phylogenetic tree structure, suggesting a common ancestor for all the viruses (Díez-Fuertes et al., 2015). Our results revealed a significant geographic dissemination of the sub-subtype A6 across the FSU countries with the lack of country-specific clades. HIV-1 sub-subtype A6 phylogenetics discovered multiple introductions of the virus to Kyrgyzstan, which are most likely associated with the intense migration between Kyrgyzstan and the neighboring countries. The origin date for sub-subtype A6 in Kyrgyzstan was estimated as June 1995. The HIV-1 effective population size of these subtypes had an initial growing phase with the further stable phase for CRF02_AG/CRF63_02A and decline phase for sub-subtype A6. CRF63_02A (formally known as CRF02_AG recombinant) was initially described in 2012 as a viral variant that caused an outbreak in the Asian part of Russia in 2006 (Baryshev et al., 2012). In our study, a very limited number of CRF63_02A sequences were detected. A small number (1.6%) of HIV-positive MSM was found in the study. Almost a half of those individuals were infected by minor HIV-1 variants (subtypes B and G, and URFs B/CRF02_AG and B/G); the remaining five MSM had sub-subtype A6 or CRF02_AG HIV-1 infection. Despite the very limited number of MSM in our study, these data indicate the mixing of HIV-1 transmissions between MSM and general population. Also, the high HIV-1 genetic diversity among MSM could potentially be one of the driving forces for further increase of HIV-1 variety in Kyrgyzstan. Data of HIV-1 prevalence among MSM in Central Asia, and particularly in Kyrgyzstan, are very limited due to criminalization of same-sex relationships (Thorne et al., 2010; Wirtz et al., 2013). However, previous studies reported HIV-1 prevalence rate among MSM at around 1% (Thorne et al., 2010; El-Bassel et al., 2013). Further studies are needed to better understand HIV-1 epidemiology and HIV-related risk behavior among this high-risk group in Kyrgyzstan. This study showed that groups of at-risk people could contribute to the HIV-1 epidemic progression by maintaining new infections within the risk group or by linking infections between different populations.

Our study has several limitations. Children and young adults were sampled more intensely than older individuals, which could have biased some of the observed numbers. In this study, samples from children and young adults were deliberately collected from those who potentially acquired HIV-1 infection during in-hospital outbreaks. Also, this study provides information on HIV-positive pregnant women in Bishkek province only. Data on pregnancy status from other provinces were not available. In this study, we detected a small number of transmission clusters sized two to four individuals due to a small sampling fraction. The study dataset represents only around 6% (555 HIV-positive study individuals of an estimated 9,200 HIV-infections in 2020 (see text footnote 1) of the total HIV-1 detected cases in Kyrgyzstan. Major DRMs were detected in 23.6% of individuals in the HIV-1 transmission clusters. The study as performed does not power to reveal whether DRMs occurred due to the therapy failure or whether originating from the transmission of the drug-resistant virus. HIV-positive individuals not linked to care were not included in the study, since only individuals who visited healthcare facilities were included.

To our knowledge, this is the first study that provides comprehensive data on the HIV-1 epidemic in Kyrgyzstan. We identified complex patterns of HIV-1 phylogenetics among the key and general populations and observed high levels of HIV-1 genetic diversity and drug resistance. Further studies are needed, accompanied by extensive public health interventions to limit HIV-1 spread and improve future HIV-1 care services.

## Data Availability Statement

The datasets presented in this study can be found in online repositories. The names of the repository/repositories and accession number(s) can be found in the article/Supplementary Material.

## Ethics Statement

The study was approved by Ethical Committee of Research and Manufacturing Association “Prevention Medicine” of Ministry of Health of Kyrgyz Republic. Written consent forms were provided by all the study individuals. For individuals 18 years old and younger, written consent forms were provided by their parent or legal guardian.

## Author Contributions

MS, AT, and NG planned and designed the study. DZ, IO, TN, MG, and VI performed HIV genotyping and collected the sequences. AT submitted the sequences. MS performed the analysis of the epidemiological data, performed the phylogenetic and phylodynamic analyses, produced the illustrations, and wrote the manuscript. NG supervised the project and edited the manuscript. IM performed the statistical analysis. UC, EN, UM, ZA, NA, AB, and UK collected the epidemiological data and assisted with sample collection at the study sites. All authors participated in the final review of the manuscript.

## Conflict of Interest

The authors declare that the research was conducted in the absence of any commercial or financial relationships that could be construed as a potential conflict of interest.

## Publisher’s Note

All claims expressed in this article are solely those of the authors and do not necessarily represent those of their affiliated organizations, or those of the publisher, the editors and the reviewers. Any product that may be evaluated in this article, or claim that may be made by its manufacturer, is not guaranteed or endorsed by the publisher.

## Footnotes

1
https://www.unaids.org/en/regionscountries/countries/kyrgyzstan

2
http://aidscenter.kg/statistika/

3
http://med.kg/images/MyFiles/KP/2018/sbornik_KP_VISH_prikaz_903_10102017.pdf

4
http://www.hiv.lanl.gov/

5
http://www.rstudio.com/

6
https://www.ncbi.nlm.nih.gov/genbank/

7
https://www.unodc.org/documents/publications/NR_Report_21.06.18_low.pdf
