# Supplementary material for: Characterization of HIV-1 Epidemic in Kyrgyzstan
Source: Front Microbiol. 2021 Oct 15;12:753675. doi: 10.3389/fmicb.2021.753675 (PMC8554114; doi:10.3389/fmicb.2021.753675)
Supplement: Supplementary file 1 [file Table_1.docx]

Supplementary Material

# Supplementary Table

Supplementary Table. Distribution of study individuals by demographic and HIV-related characteristics with the indication of gender (A) and age group (B).

A.

| **Characteristics** | **Male, n=282** | **Female, n=273** | **P** | **Adj. P** |  |
| --- | --- | --- | --- | --- | --- |
| Region, n (%) |  |  |  |  | |
| Bishkek | 98 (36.4) | 104 (41.3) | 0.41 | 1 | |
| Osh | 180 (62.1) | 161 (55.6) |  |  | |
| JAB | 4 (1.5) | 8 (3.2) |  |  | |
| Transmission mode, n (%) |  |  |  |  | |
| Heterosexual | 48 (17.1) | 167 (72.6) | **<0.01** | **<0.01** | |
| MSM | 9 (3) | 0 (0) |  |  | |
| Vertical | 41 (14.1) | 29 (10.7) |  |  | |
| PWID | 93 (33.5) | 10 (4) |  |  | |
| Nosocomial | 72 (25.3) | 47 (15.9) |  |  | |
| Unknown/No data | 19 (7.1) | 20 (7.9) |  |  | |
| HIV-1 drug resistance mutations, n (%) |  |  |  |  | |
| Yes | 81 (30.1) | 68 (24.9) | 0.39 | 1 | |
| No | 201 (69.9) | 205 (75.1) |  |  | |
| Dual- and multi-class HIV-1 drug resistance, n (%) |  |  |  |  | |
| Yes | 60 (60.6) | 39 (39.4) | 0.02 | 0.13 | |
| No | 21 (39.4) | 29 (60.6) |  |  | |
| HIV-1 subtyping, n (%) |  |  |  |  | |
| CRF02_AG | 166 (59.1) | 166 (61.5) | 0.67 | 1 | |
| A6 | 94 (33.5) | 90 (32.5) |  |  | |
| CRF63_02A | 4 (1.5) | 6 (2.4) |  |  | |
| Minor subtypes and recombinants | 18 (5.9) | 11 (3.6) |  |  | |

B.

| **Characteristics** | **Age group (years)** | | | | **P** | **Adj. P** |
| --- | --- | --- | --- | --- | --- | --- |
|  | **3-14, n=101** | **15-24, n=120** | **25-49, n=271** | **50-72, n=63** |  |  |
| Region, n (%) |  |  |  |  |  |  |
| Bishkek | 5 (5) | 17 (14.2) | 144 (53.1) | 36 (57.1) | **<0.01** | **<0.01** |
| Osh | 95 (94.1) | 103 (85.8) | 97 (35.8) | 24 (38.1) |  |  |
| JAB | 1 (.9) | 0 (0) | 8 (3.1) | 3 (4.8) |  |  |
| Gender, n (%) |  |  |  |  |  |  |
| Male | 61 (60.4) | 72 (60) | 105 (38.8) | 38 (60.3) | **<0.01** | **<0.01** |
| Female | 40 (39.6) | 48 (40) | 160 (59.1) | 25 (39.7) |  |  |
| Transmission mode, n (%) |  |  |  |  |  |  |
| Heterosexual | 1 (.9) | 8 (6.7) | 176 (65) | 30 (47.6) | **<0.01** | **<0.01** |
| MSM | 0 (0) | 3 (2.5) | 6 (2.2) | 0 (0) |  |  |
| Vertical | 43 (42.6) | 27 (22.5) | 0 (0) | 0 (0) |  |  |
| PWID | 0 (0) | 0 (0) | 72 (26.6) | 31 (49.2) |  |  |
| Nosocomial | 52 (51.5) | 67 (58.3) | 0 (0) | 0 (0) |  |  |
| Unknown/No data | 5 (5) | 15 (12.5) | 17 (6.3) | 2 (3.2) |  |  |
| HIV-1 drug resistance mutations, n (%) |  |  |  |  |  |  |
| Yes | 33 (32.7) | 40 (33.3) | 62 (23.2) | 14 (23.8) | 0.09 | 0.44 |
| No | 68 (67.3) | 80 (66.7) | 209 (76.8) | 49 (76.2) |  |  |
| Dual- and multi-class HIV-1 drug resistance, n (%) |  |  |  |  |  |  |
| Yes | 30 (90.9) | 32 (80) | 29 (46) | 8 (53.3) | **<0.01** | **<0.01** |
| No | 3 (9.1) | 8 (20) | 33 (76.8) | 6 (76.2) |  |  |
| HIV-1 subtyping, n (%) |  |  |  |  |  |  |
| CRF02_AG | 82 (81.2) | 106 (88.3) | 124 (45.8) | 20 (31.8) | **<0.01** | **<0.01** |
| A6 | 16 (15.8) | 11 (9.2) | 120 (44.3) | 37 (58.7) |  |  |
| CRF63_02A | 3 (3) | 0 (0) | 7 (2.6) | 0 (0) |  |  |
| Minor subtypes and recombinants | 0 (0) | 3 (2.5) | 20 (7.4) | 6 (9.5) |  |  |

Numbers in bold indicate statistically significant associations. Abbreviations: JAB: Jalal-Abad and Batken provinces; Adj. P: adjusted p-value; MSM: men who have sex with men; PWID: persons who inject drug; CRF: circulating recombinant form.
